# In Situ “Humanization” of Porcine Bioprostheses: Demonstration of Tendon Bioprostheses Conversion into Human ACL and Possible Implications for Heart Valve Bioprostheses

**DOI:** 10.3390/bioengineering8010010

**Published:** 2021-01-12

**Authors:** Uri Galili, Kevin R. Stone

**Affiliations:** 1 Division of Cardiology, Department of Medicine, Rush Medical College, Chicago, IL 60612, USA; 2 The Stone Clinic and Research Foundation, San Francisco, CA 94123, USA; kstonemd@stoneclinic.com

**Keywords:** heart valve bioprosthesis, anterior cruciate ligament reconstruction, porcine tendon bioprosthesis, anti-Gal antibody, anti-non gal antibody, α-gal epitope, α-galactosidase, bioprosthesis humanization

## Abstract

This review describes the first studies on successful conversion of porcine soft-tissue bioprostheses into viable permanently functional tissue in humans. This process includes gradual degradation of the porcine tissue, with concomitant neo-vascularization and reconstruction of the implanted bioprosthesis with human cells and extracellular matrix. Such a reconstruction process is referred to in this review as “humanization”. Humanization was achieved with porcine bone-patellar-tendon-bone (BTB), replacing torn anterior-cruciate-ligament (ACL) in patients. In addition to its possible use in orthopedic surgery, it is suggested that this humanization method should be studied as a possible mechanism for converting implanted porcine bioprosthetic heart-valves (BHV) into viable tissue valves in young patients. Presently, these patients are only implanted with mechanical heart-valves, which require constant anticoagulation therapy. The processing of porcine bioprostheses, which enables humanization, includes elimination of α-gal epitopes and partial (incomplete) crosslinking with glutaraldehyde. Studies on implantation of porcine BTB bioprostheses indicated that enzymatic elimination of α-gal epitopes prevents subsequent accelerated destruction of implanted tissues by the natural anti-Gal antibody, whereas the partial crosslinking by glutaraldehyde molecules results in their function as “speed bumps” that slow the infiltration of macrophages. Anti-non gal antibodies produced against porcine antigens in implanted bioprostheses recruit macrophages, which infiltrate at a pace that enables slow degradation of the porcine tissue, neo-vascularization, and infiltration of fibroblasts. These fibroblasts align with the porcine collagen-fibers scaffold, secrete their collagen-fibers and other extracellular-matrix (ECM) components, and gradually replace porcine tissues degraded by macrophages with autologous functional viable tissue. Porcine BTB implanted in patients completes humanization into autologous ACL within ~2 years. The similarities in cells and ECM comprising heart-valves and tendons, raises the possibility that porcine BHV undergoing a similar processing, may also undergo humanization, resulting in formation of an autologous, viable, permanently functional, non-calcifying heart-valves.

## 1. Introduction

A major objective in the bioengineering research of biomaterials implanted in humans is the formation of bioprostheses that will gradually convert into autologous viable functional tissues, and which conserve their biomechanical function, thereby extending their durability for life. This review describes preclinical and clinical studies of a novel method that enables the conversion of soft tissue porcine bioprostheses into human autologous functional tissue in patients with torn anterior cruciate ligament (ACL), and further discusses the potential applicability of this method to bioprosthetic porcine heart valves (BHV). The limitations of the currently used soft tissue porcine bioprostheses are exemplified in BHV implanted in young patients for the replacement of impaired heart valves. 

BHV crosslinked by glutaraldehyde are the most common bioprosthesis used in the clinic [1,2,3]. The implanted BHV have a limited durability of 10–20 years in elderly recipients (>70 year), whereas the failure rate is nearly 100% within 5 years in patients <35 years old [1,2,3,4,5]. The much lower durability of BHV in young vs. elderly recipients is the result of extensive antibody response in the young against porcine antigens, resulting in chronic inflammation that calcifies the BHV, and forms a pannus, tear, or perforation of the valve leaflets [5,6,7,8,9,10,11]. This immune response results in impairment of the BHV function and stenosis within 1–5 years post implantation of the BHV in young recipients. Due to this limited durability, young patients are implanted with mechanical valves which require constant anticoagulation therapy. In an attempt to decrease the anti-BHV immune response in young recipients, the porcine BHV underwent decellularization as part of their processing [12,13,14,15,16]. However, decellularization was found not to prevent antibody production against the porcine valve antigens [17,18,19]. Decellularization further caused substantial loss in valve stiffness, and resulted in significant extracellular matrix (ECM) disruption [18,19,20]. It has been further suggested that the impairment of porcine BHV in young recipients may be delayed by eliminating immunogenic carbohydrate antigens from the BHV, such as α-gal and Neu5Gc [1,21,22,23,24,25,26]. However, as discussed below, removal of carbohydrate antigens does not eliminate the elicited antibody production against the multiple porcine protein antigens which are immunogenic in humans. These shortcomings of BHV might be resolved if bioprostheses could be gradually converted post implantation into autologous heart valves. This process of gradual, in situ reconstruction of an animal originated bioprosthesis into a human viable, functional tissue is referred to in this review as “humanization” of the bioprosthesis, a term previously used for in vitro humanization of bone [27].

Humanization of porcine bioprostheses may be feasible if they are processed in a way that enables their gradual degradation and destruction post implantation at a pace slow enough to allow for their reconstruction by autologous cells and ECM within the recipient. In presently used BHV such degradation is minimal, because of the extensive complete crosslinking by glutaraldehyde which prevents penetration of macrophages and granulocytes beyond the surface of the implant. The pace of degradation of bioprosthetic implants may be controlled by partial (i.e., moderate), rather than complete, crosslinking of the BHV. This partial crosslinking should be further combined with the attenuation of the immune mediated rejection of porcine bioprosthesis by avoiding the activity of a natural antibody, called the natural anti-Gal antibody, and by the harnessing of elicited anti-non gal antibodies for the gradual destruction of the bioprosthesis and its subsequent humanization. These antibodies recruit macrophages that mediate the gradual degradation of the bioprosthesis, and enable infiltration of fibroblasts that align with the porcine collagen fibers “scaffold”, and secret their own ECM, including collagen fibers that replace porcine disrupted collagen fibers. The orientation and organization of the infiltrating fibroblasts within the different parts of the BHV is directed by the porcine ECM scaffold that is characteristic of each of the different parts of the BHV. Thus, it is suggested that such BHV implants may undergo humanization and convert into functional viable heart valves, consisting of cells and ECM autologous to the implant recipient. Whereas no studies testing this humanization method have been performed with BHV in experimental animal models, successful studies using the suggested method have been performed in monkeys and humans implanted with porcine bone-patellar tendon-bone (BTB) bioprostheses that replace torn ACL. The studies on the humanization of porcine BTB bioprostheses in patients with torn ACL, which are described in this review, may be regarded as a “proof of principle” for the efficacy of this method in in situ humanization of soft tissue porcine bioprostheses.

The ACL is the key stabilizer of the knee joint and is frequently torn in athletic activities. Current surgery for reconstruction of torn ACL includes grafting of autologous harvested tendon from the uninjured leg or of allogeneic cadaveric tendon. These grafting techniques have disadvantages and risks. ACL reconstruction with autologous tissue involves two surgical sites, resulting in additional incisions, increased pain, longer recovery periods, and increased morbidity. Cadaveric tendon allografts are of variable quality and the availability of those of good quality, with long term biomechanical performance for ACL reconstruction, is limited due to the scarcity of tissue from young healthy donors. In addition, allogeneic grafts carry the risk of transmission of various pathogens. Therefore, it was of interest to determine whether a porcine BTB bioprosthesis undergoing processing that diminishes rejection by the natural anti-Gal antibody, and which is partially crosslinked by glutaraldehyde, can undergo humanization in patients with torn ACL and become functional, autologous viable ACL. As described in this review, such humanization of bioprostheses is completed within ~2 years, while permanently maintaining the function of this ligament. Based on the results of humanization of BTB in replacing torn ACL, we suggest that it would be of interest to determine whether a similar processing of BHV may enable humanization of these bioprostheses in young patients with impaired heart valves.

## 2. Anti-Porcine Antibody Response in Humans Implanted with Porcine Bioprostheses

Success in humanization of porcine bioprostheses requires the selective prevention of anti-Gal antibody response to the α-gal epitope in these bioprostheses, and the harnessing of anti-non gal antibodies for mediating this humanization process. 

### 2.1. Anti-Gal Antibody

**Figure 1:**
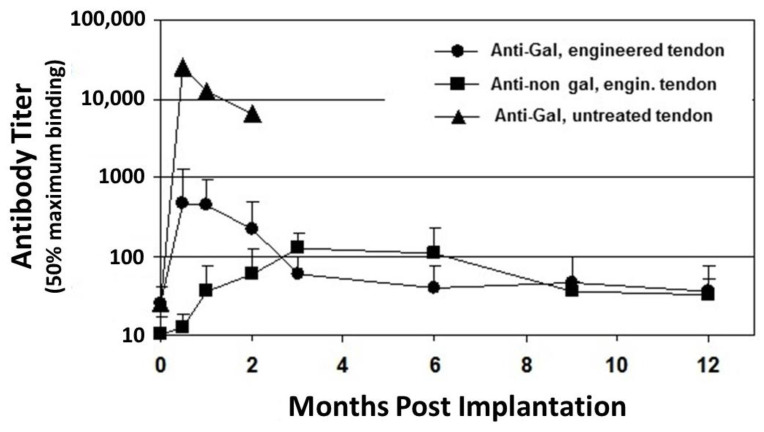
Anti-Gal and anti–non-Gal IgG titers at various time points post implantation of porcine patellar-tendon in rhesus monkeys (n = 13). Anti-Gal titers were measured by ELISA with synthetic α-gal epitopes linked to bovine serum albumin (BSA) as solid-phase antigen. Anti-non gal antibodies measured by ELISA with homogenate of unprocessed porcine patellar-tendon as solid-phase antigen and with sera that were depleted of anti-Gal. The implanted tendon was unprocessed or was a bone-patellar-tendon-bone (BTB) bioprosthesis processed by treatment with recombinant α-galactosidase and partial crosslinking with glutaraldehyde. Error bars represent standard deviation of titers (modified from [53]).

Anti-Gal is a natural antibody (i.e., antibody produced without active immunization) constituting ~1% of immunoglobulins in humans [28,29,30,31]. Anti-Gal binds specifically to a carbohydrate antigen called the α-gal epitope, with the structure Galα1-3Galβ1-4GlcNAc-R [32,33,34]. The α-gal epitope is abundantly synthesized, and presented on cells of non-primate mammals, lemurs, and New-World monkeys (10^5^–10^7^ α-gal epitopes per cell) [33,35,36]. In contrast, Old-World monkeys, apes, and humans completely lack α-gal epitopes, but all produce the natural anti-Gal antibody [33,35,37]. Anti-Gal binding to α-gal epitopes on porcine viable organ xenografts (e.g., porcine heart or kidney) causes their hyperacute rejection, in Old World monkeys and in humans, primarily as a result of anti-Gal mediated activation of the complement system that kills endothelial cells binding anti-Gal, resulting in collapse of the vascular bed of the xenograft [38,39,40,41,42,43,44]. Anti-Gal also mediates chronic rejection of xenograft cells originating in non-primate mammals. As many as 1% of circulating B cells in humans are capable of producing anti-Gal, however, most of them are quiescent [45], and those along the gastrointestinal track produce anti-Gal in response to continuous antigenic stimulation by gastrointestinal bacteria [46,47,48]. Upon exposure to α-gal epitopes on xenografts, the quiescent anti-Gal B cells are activated, resulting in extensive production of elicited anti-Gal antibody, which markedly increase titers of this antibody within 10–14 days (Figure 1).

Anti-Gal is also detrimental to porcine bioprostheses that are crosslinked by glutaraldehyde. Implantation of porcine BHV results in a marked increase in anti-Gal titers in adults [49,50,51] and in child recipients [52], because α-gal epitopes on porcine BHV activate many quiescent anti-Gal B cells to produce the antibody. The full extent of the robust activation of anti-Gal B cells by α-gal epitopes on porcine tissue was shown in rhesus monkey transplanted with unprocessed porcine patellar tendon [53]. Anti-Gal IgG titer increased by ~1000-fold, two weeks post transplantation of this tendon (Figure 1). Within two months, the grafted unprocessed porcine tendon had nearly completely disappeared because of what seems to be extensive macrophage mediated destruction of the xenograft, which was induced by the elicited anti-Gal antibody [53]. Many macrophages are recruited into the xenograft by chemotactic complement cleavage peptides, produced as a result of complement activation by anti-Gal binding to α-gal epitopes in the porcine tissue. These macrophages bind via their Fc receptors to the Fc portion of anti-Gal on the tendon cells and ECM and effectively degrade the porcine tissue to the extent that it is resorbed within two months. This anti-Gal induced chronic rejection of a porcine xenograft was also demonstrated in cynomolgus monkeys transplanted with porcine cartilage [54]. Two months post transplantation, the cartilage xenografts were filled with macrophages that degraded the tissue. However, enzymatic elimination of α-gal epitopes from these cartilage xenografts resulted in a >90% decrease (but not complete elimination) of infiltrating macrophages within these xenografts [54]. The detrimental role of anti-Gal was further demonstrated with BHV leaflets immunocomplexed with anti-Gal, which developed much more in vivo calcification than leaflets that lacked this antibody [55,56]. A 30–200 fold increase in anti-Gal titers in response to α-gal epitopes on nonprimate xenograft was also observed in patients transplanted with live porcine islet cells [57], live mouse cells [58,59], or with porcine BHV [49,50,51,52]. All these observations led to the conclusion that elimination of the α-gal epitope from the porcine tissue will contribute to the success of both xenografts and bioprostheses [1,14,15,21,22,23,24,25,26,55,56]. Such an elimination is feasible in porcine bioprostheses by enzymatic destruction of α-gal epitopes with the recombinant enzyme α-galactosidase, which cleaves the terminal galactose from the α-gal epitope (Galα1-3Galβ1-4GlcNAc-R) [15,21,22,23,24,53,54]. The remaining N-acetyllactosamine epitope (Galβ1-4GlcNAc-R) on the glycans does not bind anti-Gal. Alternatively, bioprostheses may be prepared from pigs that have been engineered to lack α-gal epitopes by disruption of the α1,3galactosyltransferase gene (*α1,3GT* gene also called *GGTA1*), which codes the enzyme α1,3galactosyltransferase that synthesizes α-gal epitopes [25,26,60,61].

### 2.2. Anti-Non Gal Antibodies

Human anti-non gal antibodies which react against porcine antigens in BHV consist of the natural anti-carbohydrate anti-Neu5Gc antibody and the elicited antibodies against the multiple porcine protein antigens. Anti-Neu5Gc is naturally produced in humans against the sialic acid, N-5-glycolyl-neuraminic acid (Neu5Gc) [62,63,64]. Neu5Gc is synthesized in all apes, Old World monkeys, and many non-primate mammalian species, but is absent in humans [65,66,67]. Since Neu5Gc is synthesized in pigs, as well as in many other nonprimate mammals, the natural anti-Neu5Gc antibody is considered to be detrimental to live xenografts in humans [68,69]. For this reason, genetically engineered pigs, lacking both α-gal epitopes and Neu5Gc, have been generated [25,26]. However, in the clinical trials with porcine tendon bioprostheses described in this review, anti-Neu5Gc antibody in human serum was not found to be detrimental to the humanization of these bioprostheses. 

**Figure 2:**
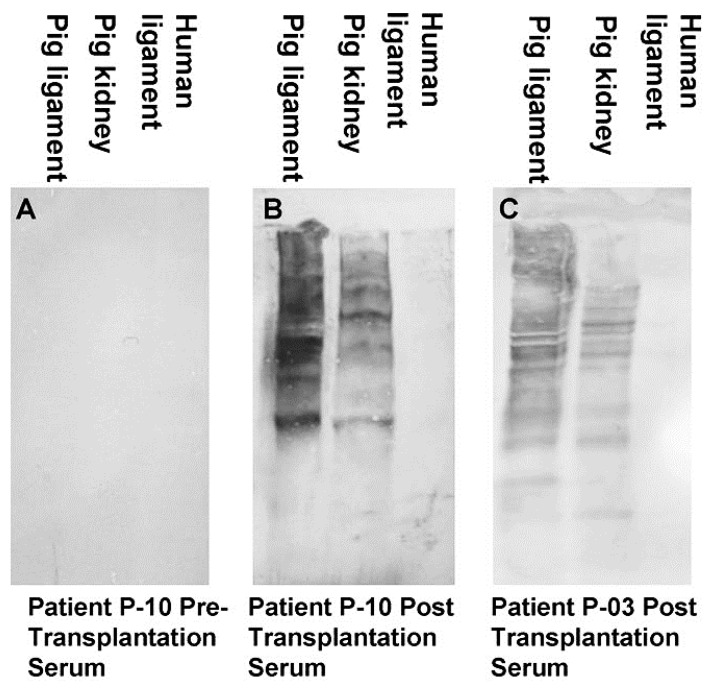
Anti-non gal antibody analysis by Western blots with porcine patellar-tendon and kidney proteins, or human patellar-tendon proteins separated by sodium dodecyl sulfate-polyacrylamide gel electrophoresis (SDS-PAGE). (**A**) Pre-implantation serum of patient P-10. (**B**) Serum of patient P-10, six months post-implantation of porcine BTB. (**C**) Serum of patient P-03, six months post-implantation of porcine BTB. In this analysis, the sera were diluted 1:10, and depleted of anti-Gal by adsorption on glutaraldehyde fixed rabbit red blood cells (RBC) (modified from [71]).

A second group of anti-non gal antibodies are the de novo produced antibodies against the multiple porcine immunogenic xeno-proteins of bioprostheses implanted in humans. Most proteins in non-primate mammals are immunogenic in humans because of the ~3–40% difference in their amino acid sequences in comparison to homologous proteins in humans [58,70]. These antibodies are shown in Figure 2, in which human anti-non-gal antibodies to porcine BTB proteins were studied by Western blot analysis. BTB proteins were separated by electrophoresis in polyacrylamide gel, blotted on nitrocellulose that was subsequently incubated with sera (diluted 1:10) depleted of the anti-Gal antibodies by adsorption on glutaraldehyde fixed rabbit red blood cells (RBC) [71]. No anti-non gal antibodies binding to porcine BTB proteins were detected in pre-implantation serum (Figure 2A). However, sera obtained 6 months post implantation contained multiple antibodies that bound to many porcine tendon proteins, resulting in a partial overlap between bands of antibodies bound to proteins of close size (Figure 2B,C). Some of the porcine proteins binding these antibodies were also observed in porcine kidneys. Anti-non-gal antibodies were highly specific to porcine antigens, and did not bind to human BTB proteins (Figure 2B,C). Production of anti-protein, anti-non gal antibodies was also observed in sheep transplanted with decellularized porcine valve leaflets [18], and in humans injected intraperitoneally with mouse xenograft cells, in the course of an experimental cancer therapy study [58]. Similar to anti-Gal, anti-non-gal antibodies binding to bioprosthesis cells and ECM are likely to activate the complement system, chemotactically recruit macrophages by complement cleavage peptides, such as C5a, and induce gradual destruction of the implant. However, as described below, the destruction of porcine bioprosthesis implants by the anti-non-gal antibodies can be controlled to occur at a pace that is slow enough to enable the harnessing of these antibodies for mediating humanization of the implant by its gradual reconstruction into a functional autologous viable tissue.

## 3. Hypothesis on Humanization of Porcine Bioprostheses 

We hypothesized that the process of humanization of soft tissue porcine bioprosthesis implants may involve the physiologic mechanisms of repair and regeneration observed in wound healing [72,73,74]. Within few days post injury, pro-inflammatory macrophages migrate into the injury site, and debride it of dead cells and degraded ECM. Subsequently, pro-reparative macrophages in the debrided injury site secrete cytokines that orchestrate the neo-vascularization and recruitment of cells which repair the injury site. The reconstruction of human ACL by grafting of autologous or allogeneic cadaveric tendons by a process called “ligamentization” [75,76,77] was found to include similar elements of the healing processes as those in wound repair. In the ligamentization process, the fibroblasts within the implant become necrotic, because of ischemia, and macrophages infiltrating the implant debride the necrotic tissue and induce neo-vascularization of the implant. Fibroblasts of the recipient repopulate the implant, and align with its collagen fibers “scaffold”. These fibroblasts secrete collagen and other matrix proteins, ultimately resulting in gradual remodeling the implant into an autologous tissue, with characteristic ACL structure and function. 

**Figure 3:**
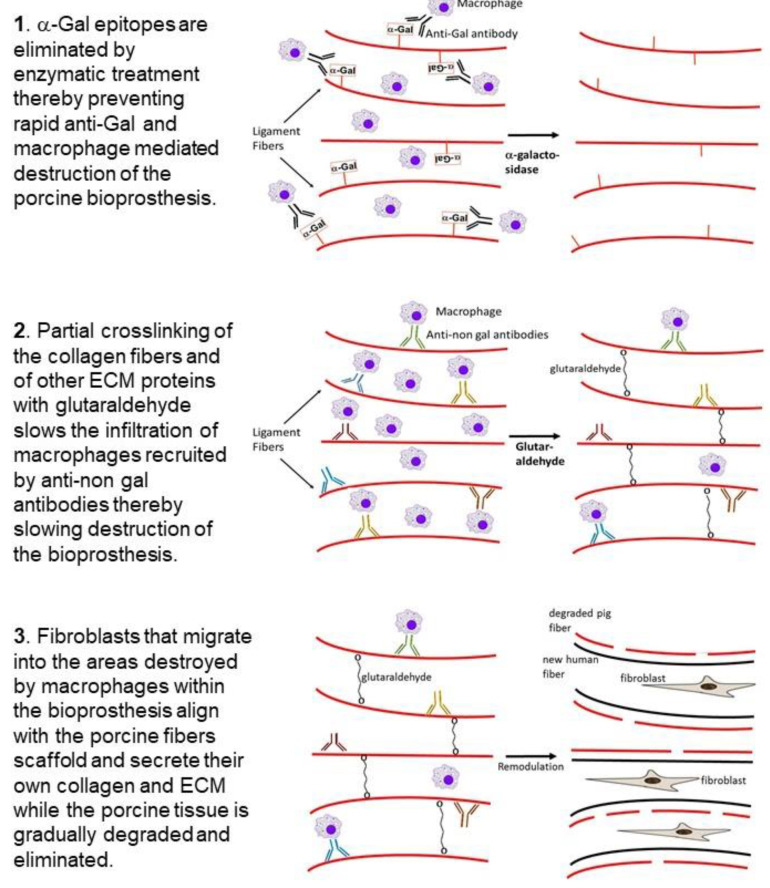
Hypothesis on humanization of porcine BTB or BHV bioprostheses implanted into patients with torn ACL or with impaired heart valve, respectively: **Stage 1.** Elimination of α-gal epitopes from the bioprosthesis by incubation with recombinant α-galactosidase prevents accelerated destruction by anti-Gal and by the macrophages it recruits. **Stage 2.** Partial crosslinking with glutaraldehyde creates “speed bumps” that slow macrophage infiltration following recruitment of the macrophages into the bioprosthesis by anti-non-gal antibodies binding to many porcine protein antigens. Colors of the antibody molecules vary because of different specificities of anti-non-gal antibodies. The macrophages bind via Fc-receptors to the Fc “tail” of anti-non-gal IgG molecules that interact with the porcine antigens of the ECM, and of non-viable cells in the implanted bioprosthesis. **Stage 3.** The porcine tissue is gradually degraded by the infiltrating macrophages. Fibroblasts that follow the macrophages align with the porcine collagen fibers scaffold and secrete their collagen and other ECM components. This concomitant destruction and reconstruction (remodulation) results in humanization of the bioprosthesis by gradual replacement of the porcine tissue with autologous permanently functional ACL or heart valve.

We hypothesized that bioprosthesis implants such as porcine BTB, and possibly BHV, may be subjected to similar processes of degradation and reconstruction as those observed in the ligamentization of autologous or allogeneic tendons replacing torn ACL. However, in view of the extensive anti-Gal immune response following exposure of the immune system to porcine α-gal epitopes, we assumed that humanization of porcine soft tissue bioprostheses requires elimination of α-gal epitopes, to prevent binding of anti-Gal to implanted bioprostheses (**Stage 1** in Figure 3). If this is not prevented, natural and elicited anti-Gal antibodies binding to the bioprosthesis are likely to result in an extensive chronic inflammatory reaction that accelerates destruction of the bioprosthesis, and thus prevent appropriate reconstruction of the implant into an autologous ACL or heart valve. We further hypothesized that bioprostheses should be only partially crosslinked with glutaraldehyde (or other crosslinkers), instead of the complete crosslinking as presently performed with porcine BHV. The partial crosslinking is expected to slow macrophage infiltration, thus decreasing the pace of bioprosthesis degradation, and thereby enabling neo-vascularization and migration of fibroblasts. These fibroblasts follow the macrophages and gradually reconstruct the degraded parts of the bioprosthesis. Crosslinking glutaraldehyde molecules function as “speed bumps” that slow, but do not prevent, macrophage infiltration (**Stage 2**). The complete crosslinking, due to prolonged immersion of the bioprosthesis in glutaraldehyde, presently performed with porcine BHV, blocks any macrophage infiltration into the bioprosthesis, and thus prevents the gradual degradation which is required for the humanization process. 

One of the main factors inducing macrophage infiltration into the bioprosthesis is anti-non gal antibodies against porcine protein antigens, which are continuously produced for as long as there are porcine antigens in the bioprosthesis (Figure 2). Binding of these antibodies to the ECM, and to the dead crosslinked cells within the bioprosthesis, results in complement activation [17]. One of the byproducts of this activation is the formation of complement cleavage peptides (e.g., C5a) that induce continuous chemotactic recruitment of macrophages into the bioprosthesis. The infiltrating macrophages bind via their Fc receptors to anti-non gal antibodies immunocomplexed to the porcine ECM and cells and cause the slow degradation of the bioprosthesis. The macrophages further debride the bioprosthesis and induce gradual neo-vascularization by the vascular endothelial growth factor (VEGF) they secrete. Fibroblasts infiltrating via the newly formed blood vessels, align with the porcine ECM collagen fibers scaffold, and secrete their own collagen fibers (**Stage 3**). Overall, the gradual degradation of the porcine tissue within bioprostheses by macrophages recruited by anti-non gal antibodies, and the concomitant replacement of the degraded areas with human fibroblasts and ECM were hypothesized to result in the humanization of implanted bioprostheses into autologous biomechanically functional viable tissue, and to prevent short or long term impairment of the bioprosthesis function and calcification. As described below, this hypothesis was tested with porcine BTB bioprostheses initially in monkeys, and subsequently in patients in whom such bioprostheses were implanted for replacement of torn ACL.

## 4. Processing of Porcine Patellar-Tendon into Bioprostheses, and Pre-Clinical Studies in Monkeys 

**Figure 4:**
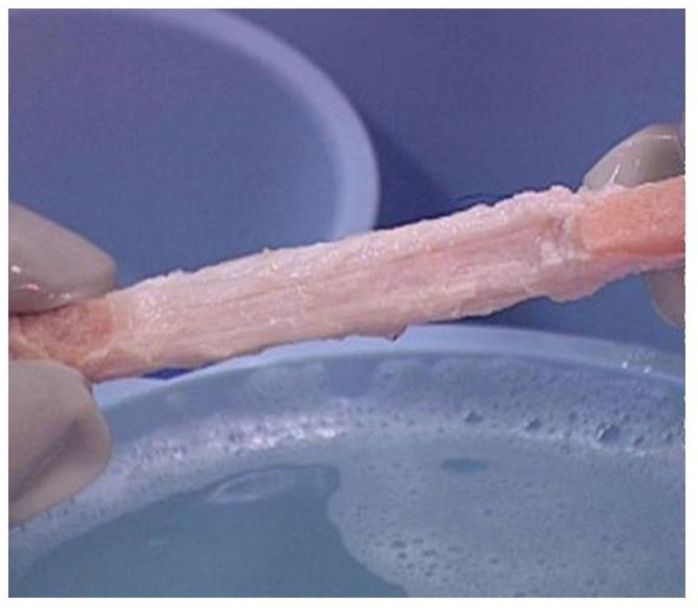
Bioprosthesis prepared from porcine bone-patellar-tendon-bone (length of ~10 cm and width of ~1 cm) for reconstructing torn ACL in humans. Note the two bone-plugs of the patella and tibia bones.

Porcine patellar-tendons and the attached patellar and tibial bone-plugs (Figure 4) were processed to remove α-gal epitopes by incubation of the tendon for 12 h in recombinant (r)α-galactosidase solution [53,71]. Tendons may be of various sizes according to the age of the pig. The complete removal of α-gal epitopes was confirmed by an ELISA Inhibition Assay [54], displaying no binding of the monoclonal anti-Gal antibody M86 [78] to the homogenate of the treated tendon [71]. The patellar-tendons were washed and partially crosslinked by incubation in 0.1% glutaraldehyde for 12 h. Subsequently, these processed tendons (referred to as BTB bioprostheses) were washed, and residual active aldehyde groups of glutaraldehyde were blocked with 0.1 M glycine. 

The optimal concentration of 0.1% glutaraldehyde used for partial crosslinking was determined empirically by incubation for 12 h of α-galactosidase treated patellar-tendon specimens in solutions containing glutaraldehyde at various concentrations, then washing and blocking of free aldehyde groups with glycine. These tendon specimens were implanted in the suprapatellar pouch of rhesus monkeys. The implants were explanted after 2 months, and their histopathology evaluated. The optimal glutaraldehyde concentration for partial crosslinking was determined as the concentration that subsequently enabled infiltration of macrophages to the extent that they occupied 20–30% of the implant. The crosslinked tendons were further preserved in 0.1 M glycine, and not in glutaraldehyde, to prevent additional crosslinking. The BTB bioprostheses were stored frozen after low level (17.8 kGy) e-Beam irradiation for final sterilization. In vitro stress tests indicated that this processing of the porcine tendons did not affect their biomechanical characteristics [53]. It should be noted that the optimal concentration of glutaraldehyde has to be determined empirically for each type of tissue, to ensure the appropriate macrophage infiltration rate in soft tissues that may contain cellular and ECM components at concentrations that differ from those in tendons. 

A pre-clinical study of BTB bioprostheses implantation was performed in 20 rhesus monkeys, in which the safety and efficacy of the method were evaluated [53]. The ACL in the monkeys was removed and reconstructed by using treated porcine BTB implants, or allograft controls. Animals were stratified into 2-, 6-, and 12-month post implantation cohorts. Porcine BTB bioprostheses and rhesus patellar-tendon allografts were found to be incorporated by the host as functional ACL. There was no indication of toxicity with any of the bioprostheses. Both porcine BTB and rhesus tendon allografts revealed gradual host cellular infiltration and collagen remodeling similar to the ligamentization process observed in humans grafted with autologous or allogeneic (cadaveric) patellar tendon. Tensile tests of the strength of explanted transplants at the various time-points, demonstrated similar functional integration of allografts and treated porcine BTB reconstructions, and a similarity in gait between the two groups [53].

Despite the elimination of α-gal epitopes by recombinant (r)α-galactosidase, titers of anti-Gal were elevated by ~30 fold, 2–4 weeks post implantation, as measured in ELISA assays with synthetic α-gal epitopes linked to albumin as solid-phase antigen (Figure 1) [53]. This elicited anti-Gal response occurred because the of stimulation of the immune system to increase production of anti-Gal by α-gal epitopes on porcine RBC and bone marrow cells encased in the small cavities of the bone-plugs. The rα-galactosidase cannot reach these cells. However, α-gal epitopes on cells released from the bone-plugs in the course of their remodeling activate quiescent anti-Gal B cells to produce increased amounts of anti-Gal for a period of 2–10 weeks post implantation (Figure 1). The subsequent decrease in anti-Gal production, to a level close to that observed in the pre-implantation serum, suggests that the porcine bone-plugs underwent near complete remodeling into autologous monkey bone, devoid of porcine cells, within ~3 months post implantation (Figure 1). Accordingly, analysis of changes in anti-Gal titers post implantation of this bioprosthesis in humans can provide information on the extent of humanization of the bone-plugs in implanted patients at various time points [71]. The elevation in anti-Gal activity in monkeys implanted with processed porcine BTB further supports the assumption that if α-gal epitopes are not removed from the BTB, the increased anti-Gal activity elicited by α-gal epitopes on soft tissue, and on RBC released from the bone cavities, will reach very high levels, and thus destroy the bioprosthesis prior to achieving appropriate humanization of the implant.

ELISA assays for anti-non gal antibody activity could provide information on the extent of replacement of the porcine bioprostheses with autologous ACL monkey tissue at various time points. In these assays the solid-phase antigen was porcine tendon homogenate, and the sera assayed were depleted of anti-Gal (by adsorption on glutaraldehyde fixed rabbit RBC, which present multiple α-gal epitopes) prior to the assay. Anti-non gal antibody titers peaked 3–6 months post transplantation, and subsequently decreased with the increased replacement of porcine tissue with the monkey fibroblasts and the ECM they produced (Figure 1). Anti-non gal antibody activity did not return to the pre-implantation level, even at 12 months post implantation, suggesting that not all porcine soft tissue was eliminated at that time point. As described below, near complete replacement of porcine BTB with human tissue in implanted patients, was observed by the anti-non gal ELISA assay ~2 years post implantation.

## 5. Implantation of Porcine BTB Bioprosthesis in Patients with Torn ACL 

The studies in monkeys indicated that treatment with porcine BTB bioprosthesis is safe and results in remodeling and regeneration of the implant into a functional autologous monkey tissue, as hypothesized in Figure 3. Thus, the study of possible humanization of porcine BTB bioprostheses progressed to a clinical trial, performed in patients with torn ACL. This clinical trial was a non-randomized FDA and Institutional Review Board (IRB) approved Phase 1, single-center feasibility study, and included 10 consenting subjects [71]. The study group was a highly active athletic subject population. The average age was 41 years (range of 21 to 51). In each of the patients, the damaged ACL was replaced with a porcine BTB bioprosthesis. The bone-plugs of the bioprosthesis were fixed to drilled femoral and tibial tunnels with interference fit screws. Patients underwent periodic clinical examinations, radiographic and MRI examinations, and blood and urine analysis for each subject for a two-year period following surgery.

**Figure 5:**
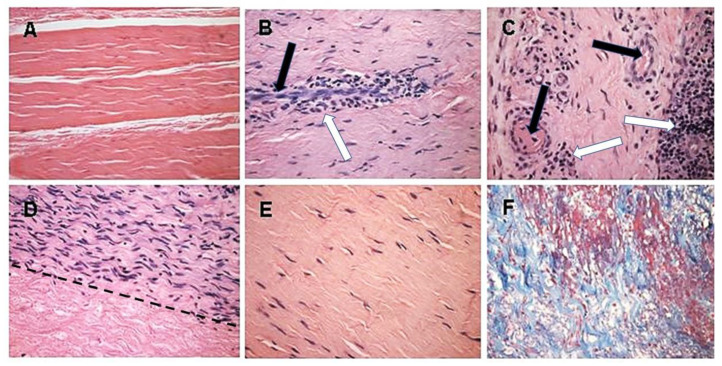
Histopathology demonstrating humanization stages in patients with implanted porcine BTB bioprostheses. Black arrows: blood vessels, white arrows: macrophages. (**A**) Pre-implantation porcine BTB bioprosthesis. (**B**) Infiltration of macrophages into the implanted bioprosthesis by extravasation. Elongated cells are endothelial cells of a blood vessel. (**C**) Vascularization of the implanted BTB in a region near macrophage infiltrates. (**D**) Repopulation of a section of the bioprosthesis by the recipient’s fibroblasts that aligned with the porcine collagen fiber scaffold (above the dashed line). Porcine collagen fibers and no cells, seen under the dashed line. (**E**) An advanced stage of humanization, with repopulating fibroblasts secreting their own ECM. (**F**) De novo produced collagen fibers, stained blue in Mason-trichrome staining. H&E, (×200) (modified from [71]).

Of the six evaluable subjects, five presented with functional humanized ACL at the 24-month post-operative time-point and satisfied all study success criteria including functional return to a high level versus the unoperated knee at 12 and 24 months after surgery. Athletes undergoing this surgery returned to regular training activity within 6–12 months. In all these patients, the humanized ACLs have continued and are continuing to function for ~17 years, and thus seem to be permanently functional. The sixth evaluable subject presented with tibial bone-plug loosening at 15-months post ACL reconstruction, had the implant removed, and was grafted with an allograft patellar tendon. The remaining four patients were non-evaluable subjects, who ruptured their porcine BTB bioprostheses due to sport injuries within the first-year post implantation, in accidents that usually cause the rupture of autologous ACL, as well. Their implants were explanted in secondary surgical interventions. Histologic examination of the explanted porcine BTB implants in the four non-evaluable subjects provided insight into the cellular events within the bioprosthesis in course of its humanization into an autologous ACL (Figure 5) [71]. 

**Figure 6:**
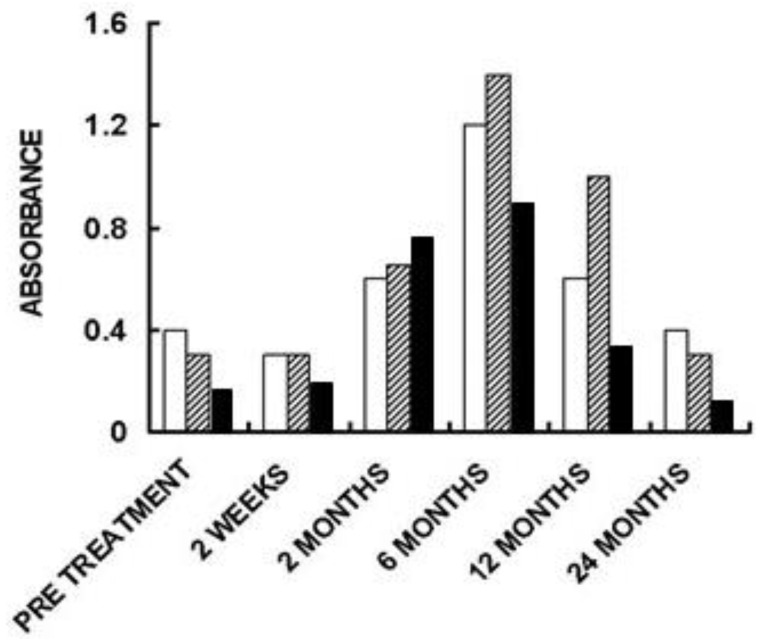
Anti-non gal IgG antibody response in three of the patients implanted with processed porcine BTB bioprosthesis for the reconstruction of torn ACL. Anti-non gal antibody activity at various time-points post-implantation was determined with anti-Gal depleted sera, by ELISA. Homogenate of fragmented porcine tendon was used as solid-phase antigen. The figure describes antibody binding at serum dilution of 1:640 (based on data from [71]).

The initial recruitment of macrophages (illustrated in **Stage 2** of Figure 3) and the start of neo-vascularization, which enables additional infiltration of macrophages, are shown in Figure 5B, in which endothelial cells of a small blood vessel are surrounded by infiltrating mononuclear cells, many of which are macrophages. The continuing migration of macrophages through the blood vessels is further shown in Figure 5C. The right section of this figure demonstrates an area with a high concentration of the infiltrating macrophages. The blood vessels also enable infiltration of the recipient’s fibroblasts, which align with the porcine collagen fibers scaffold (Figure 5D). This figure suggests that the humanization process occurs in different stages at various areas of the implant. In the upper half of the figure, multiple fibroblasts align with the porcine collagen fibers scaffold, whereas in the lower part, this scaffold is devoid of infiltrating cells. The aligned infiltrating fibroblasts secrete their own collagen fibers, and thus humanize the porcine patellar-tendon into an autologous functional, viable ACL (Figure 5E). The newly formed collagen fibers are stained blue by Mason-trichrome staining (Figure 5F). The blood vessels in Figure 5B,C are likely to be the result of neo-vascularization, since the porcine blood vessels were crosslinked by glutaraldehyde, and no anastomoses were made between the recipient blood vessels and the implanted bioprostheses. Overall, the neo-vascularization and macrophage infiltration observed in Figure 5B,C, the infiltrating fibroblasts in Figure 5D, and the newly formed collagen fibers in Figure 5F, all strongly suggest the occurrence of an active humanization process within the first year post-operatively. This suggestion is supported by the observation of the peak anti-non gal antibody production at 6 months post-operatively (Figure 6), implying ongoing degradation of the bioprostheses at that time.

The humanization of porcine BTB into autologous ACL appears to complete within ~2 years, as indicated by the anti-non gal antibody production at various time-points. The titer of these antibodies in pre-implantation sera is usually very low (~1:20) and reflects a background level (Figure 6). At the low level of pre-implantation anti-non gal antibody activity, no significant antibody binding to porcine tendon proteins was observed in Western blots (Figure 2). Anti-non gal antibody production, as measured by ELISA with homogenate of porcine tendon as solid-phase antigen, and with sera depleted of anti-Gal, peaked at ~6 months post-implantation (Figure 6). This peak anti-non gal antibody activity reflects the immune response to the multiple porcine antigens released from the porcine BTB that is gradually degraded by macrophages and is further shown in Figure 2. After 12 months, anti-non gal antibody titers decreased because of diminishing amounts of released porcine antigens. By 24 months, this antibody production decreased to a level that was within the range of the pre-implantation level, because of diminished, or absence of stimulating porcine antigens (Figure 6). This absence of anti-non gal antibodies at 24 months, strongly suggests that most or all of the original porcine tissue was replaced by permanently functioning human ACL tissue, thus completing the humanization process. 

Porcine BTB bioprostheses processed for elimination of α-gal epitopes and partial crosslinking, as described above, were also used in an international double blinded, randomized controlled clinical trial in clinical centers in Italy, Denmark, Belgium, Spain, the Netherlands, and South Africa, for the reconstruction of torn ACL [79]. That study was initiated ~10 years ago with a second group of patients that included 61 subjects with ruptured ACL, of which 32 were grafted with cadaveric allografts and 29 were implanted with porcine BTB bioprostheses. The processing of these bioprostheses was the same as that of the bioprostheses described above, and in Figure 5 and Figure 6 [53,71], but included an additional step of decellularization prior to treatment with rα-galactosidase. Six additional subjects in the BTB bioprosthesis implanted group got a deep infection in the bioprostheses, attributed to a water-based pathogen (*Ralstonia pickettii*) bioprostheses contamination that occurred during the processing. By changing the water filter from 0.2 μm to 0.05 μm, this contamination was prevented in subsequent processed bioprostheses [79]. Similar to the patients in the first group described above [71], the patients in the second group [79] presented with functional reconstructed ACL at the 24-month post-operative time-point. Moreover, at 24 months, functional performance assessment in the bioprosthesis implanted subjects satisfied all study success criteria, and did not reveal significant functional performance differences between subjects implanted with the BTB bioprosthesis and those with cadaveric allograft. In addition, anti-Gal production increased above the pre-surgery level at 2 weeks post surgery, and returned to the natural level after ~12 months. Anti-non gal antibody production peaked at 3–6 months at 100 fold the background level, and subsequently decreased back, close to the background level after 24 months [79]. Taken together, the lack of differences in functional performance between recipients of the porcine BTB bioprosthesis and the recipients of cadaveric allograft tendons, and the return of anti-Gal and anti-non gal antibody levels close to the pre-surgery level, strongly suggest that in the second group of bioprosthesis recipients, the implants also underwent humanization into autologous, functional, viable ACL. These humanized ACL have continued to function with no failure for >8 years post surgery (personal communication). The similarities in results between the study of patients in the first group [71] and those in the study of the second group [79] further suggest that the addition of the decellularization step is not required for successful humanization of the porcine bioprosthesis into viable human tissue. 

Overall, the studies above imply that elimination of α-gal epitopes and partial crosslinking of the porcine BTB to slow macrophage infiltration and degradation of the implant, enable neo-vascularization, fibroblasts infiltration, and alignment with the porcine collagen fiber scaffold. Continuous degradation of this scaffold by macrophages and its concomitant replacement with human fibroblasts, collagen fibers and other ECM components, result in humanization of the bioprosthesis into viable and permanently functional autologous ACL. 

## 6. Potential Translation of ACL Studies to Porcine BHV Implants 

This section is mostly speculative, and draws possible analogies between the structures of tendons and heart valves in order to suggest that humanization of BHV should be studied by processing these bioprostheses according to the processing of porcine BTB, described above. The tendon and heart valve are essentially comprised of collagen, elastin, proteoglycans and the fibroblasts producing these ECM components [2,80,81]. However, the collagen fibers are at a variety of orientations and concentrations in various parts of the heart valve, compared to their uniform longitudinal orientation and their similar concentration in tendons and ligaments. In addition, the luminal surface of the heart valve is covered with endothelial cells, which are absent on the surface of tendons and ligaments. Moreover, the concentration of fibroblasts and their orientation are much less uniform in the heart valve leaflets and fibrous ring than that in the tendon. Thus, it remains to be determined whether the collagen fibers scaffold at various parts of the BHV can direct the orientation of the infiltrating fibroblasts for restoring the appropriate structure and function of the heart valve, similarly to the restoration observed above in the humanized ACL. All these assumptions will have to be studied in appropriate experimental models. In the absence of porcine BHV humanization studies, this section discusses several theoretical aspects regarding the future processing of porcine BHV for evaluating the possible humanization of these bioprostheses.

### 6.1. Removal of the α-Gal Epitope from the Porcine BHV

Porcine heart valves were found to present multiple α-gal epitopes [21,24,82]. Accordingly, patients implanted with porcine BHV were found to display increased titers of anti-Gal [49,50,51,52]. Removal of α-gal epitopes from porcine BHV will prevent increased production of anti-Gal, which may be detrimental to the humanization process, because of natural and elicited anti-Gal-mediated accelerated degradation of the bioprosthesis. The feasibility of α-gal epitope elimination from the porcine heart valve was indeed demonstrated by the use of rα-galactosidase of plant or microbial sources and produced in various expression systems [21,22,23,24,53,71,83]. Confirmation for the effective removal of α-gal epitopes can be achieved by the ELISA Inhibition Assay, which measures the removal of these epitopes on homogenates of the treated tissue. In the absence of α-gal epitopes on the homogenate tissue fragments, no binding of the monoclonal anti-Gal antibody M86 to these fragments was detected [54,71,78]. An alternative possibility is the use of heart valves harvested from genetically engineered pigs lacking α-gal epitopes due to disruption of the *α1,3GT* gene (i.e., knockout pig for the *α1,3GT* gene *GGTA1*) [25,26,55,56,60,61]. In addition, these knockout pigs may be considered as an appropriate initial experimental animal model since they produce the natural anti-Gal antibody, like humans [84,85,86]. If this model is to be used, the donor of the BHV should be from a different evolutionary lineage (to simulate pig/human discordance), and the studied pigs should lack natural anti-non gal antibodies against the donor antigens.

### 6.2. Glutaraldehyde Crosslinking of the Porcine BHV 

Partial crosslinking by glutaraldehyde is performed to prevent rapid degradation of the BHV and to enable the slow infiltration of macrophages, which mediate gradual degradation. It is important to identify the optimal concentration of glutaraldehyde for this purpose. This can be determined empirically, as described for the porcine patellar-tendon [53]. As indicated above, the concentration and orientation of fibroblasts differ in the leaflets from that in the fibrous ring. It remains to be determined whether the different organization and structure of the ECM in the leaflets and fibrous ring can appropriately direct the humanization of the BHV implant. This objective of the reconstruction of implants by autologous cells and ECM has also been the goal of studies with decellularized porcine BHV [14,15,18]. Decellularization was found, however, to cause disruption of the ECM within porcine heart valve [20] and early failure of the implant [19]. Since processing of BHV by partial crosslinking does not involve immersion of the heart valve in a detergent for achieving decellularization, the biomechanical integrity of BHV described in this review may be higher than that of decellularized BHV during the in situ humanization process. Due to the constant exposure of the BHV implant to blood flow, determining the optimal glutaraldehyde concentration should be performed by evaluating macrophage infiltration under conditions that simulate the blood flow through the valve with leaflet movement. This may be feasible in a primate model in which the heart valve is replaced by porcine BHV, devoid of α-gal epitopes and subjected to 12 h crosslinking by various concentrations of glutaraldehyde. It should be noted that in many of the bound glutaraldehyde molecules, one of the two aldehyde “arms” may remain free and cause toxicity and calcification post-operatively by binding amino groups on various cells and proteins. In order to block these free aldehyde groups, and thus decrease subsequent calcification, crosslinked BHV have been treated with amino group presenting agents such as monosodium glutamate [87] or amino oleic acid [88]. In the method described here, the crosslinked bioprosthesis is immersed in glycine solution which provides the amino groups required for blocking the free arms of glutaraldehyde. In addition to various blocking agents, several crosslinking agents have been evaluated for use in BHV processing, including phytic acid [89], polyepoxy compounds [90,91], and carbodiimide [92,93]. In all these methods, as well as with glutaraldehyde crosslinked BHV, the implanted bioprosthesis functions as a dead tissue prone to calcification and disruption of the collagen fibers, with no repair [94]. In contrast, the method studied above for humanization of porcine tendon bioprostheses may allow for reconstruction of the porcine BHV into a viable, autologous valve that functions permanently, as it undergoes repair by infiltrating fibroblasts. The cellular and matrix components, including resurfacing endothelium may further avoid thrombotic complications, because they are of autologous origin. Under such circumstances, it would be of further interest to determine whether partial application of alternative crosslinkers instead of glutaraldehyde may improve the efficacy of the humanization process. 

### 6.3. Monitoring Humanization of the Implanted Porcine BHV 

In addition to the standard imaging methods for evaluating the function of the porcine BHV, it may be possible to monitor the extent of porcine tissue humanization into autologous human valve tissue by measuring anti-non gal antibody production against porcine antigens in the BHV. Based on the humanization studies with porcine tendon bioprosthesis [71], it is expected that the titer of anti-non gal antibodies will increase to a plateau for several months, and subsequently decrease because of decreasing amounts of immunizing porcine antigens. Completion of the humanization process will be indicated by a return of anti-non gal antibody titer to the pre-implantation level, due to elimination of porcine antigens. 

If remodeling and regeneration of porcine BHV in a primate model is ultimately found to be successful, clinical trials with such BHV may be considered. Success in such clinical trials may enable the use of porcine BHV in young individuals, and thus negate the need for constant use of anticoagulation therapy, which is currently required for implanting mechanical valves. Hypothetically, stentless porcine BHV, which undergo humanization may further enable their use in children, in whom humanized porcine BHV may increase in size with the growth of patients. In addition, the use of porcine BHV that humanizes may solve in all age groups the current problem of leaflet sagging, because of disruption of crosslinked collagen fibers [94]. The live recipient’s fibroblasts in the humanized BHV will provide intact collagen fibers to replace disrupted fibers. In addition to the use of porcine heart valves as BHV, many BHV are made of bovine pericardium which contains cellular and matrix components of dense fibrous tissue, similar to those in the tendon. Thus, it is possible that the valve processing method of α-gal epitopes elimination and partial crosslinking by glutaraldehyde may be considered for humanization studies of BHV made of bovine pericardium, as well. 

## 7. Conclusions 

Partially crosslinked porcine bone-patellar-tendon-bone (BTB) bioprostheses, devoid of α-gal epitopes and implanted in patients with torn ACL, undergo humanization into autologous, viable, permanently functional ACL. In that process, anti-non gal antibodies contribute to the recruitment of macrophages that infiltrate into the implanted bio- prostheses. This infiltration is slowed, but not prevented, by partial crosslinking with glutaraldehyde molecules that function as “speed bumps” within the bioprosthesis. The infiltrating macrophages induce neo-vascularization, which enables recruitment of many more macrophages that degrade and debride the bioprosthesis with the help of anti-non gal antibodies binding to the BTB. Fibroblasts, following the recruited macrophages, align with the porcine collagen fibers scaffold and secrete their own collagen and other ECM components. The humanization process, which includes gradual degradation of the bioprosthesis and the concomitant replacement of the destroyed porcine tissue with human fibroblasts and ECM, is completed within ~2 years, and results in the formation of an autologous ACL that conserves permanently the biomechanical function even in athletic patients. 

Porcine bioprostheses of heart valves (BHV) contain ECM and cellular components similar to those in tendons. Thus, studies on the possible humanization of BHV implants that are processed by elimination of α-gal epitopes and partial crosslinking by glutaraldehyde, should be considered. Successful conversion of such porcine or bovine BHV into viable, autologous, functional heart valves in experimental animal models may be followed by studies on BHV replacing impaired heart valves in young patients. Presently, these patients are implanted only with mechanical heart valves that require anticoagulation therapy. In addition, porcine, bovine, or equine (and possibly other mammalian) dermis, intestinal submucosa, pericardium, urinary bladder, blood vessels, ligaments, and other soft tissues processed to lack α-gal epitopes, and which are partially crosslinked, should be considered for studying as bioprostheses with high biomechanical integrity, and which undergo gradual humanization for conversion into functioning autologous viable tissues. 

## Data Availability

The data described in this review were previously published in quoted original papers available on PubMed.
